# *Tomato Spotted Wilt Virus* Benefits a Non-Vector Arthropod, *Tetranychus Urticae*, by Modulating Different Plant Responses in Tomato

**DOI:** 10.1371/journal.pone.0075909

**Published:** 2013-09-18

**Authors:** Punya Nachappa, David C. Margolies, James R. Nechols, Anna E. Whitfield, Dorith Rotenberg

**Affiliations:** 1 Indiana University-Purdue University Fort Wayne, Fort Wayne, Indiana, United States of America; 2 Department of Entomology, Kansas State University, Manhattan, Kansas, United States of America; 3 Department of Plant Pathology, Kansas State University, Manhattan, Kansas, United States of America; Portland State University, United States of America

## Abstract

The interaction between plant viruses and non-vector arthropod herbivores is poorly understood. However, there is accumulating evidence that plant viruses can impact fitness of non-vector herbivores. In this study, we used oligonucleotide microarrays, phytohormone, and total free amino acid analyses to characterize the molecular mechanisms underlying the interaction between Tomato spotted wilt virus (TSWV) and a non-vector arthropod, twospotted spider mite ( *Tetranychusurticae* ), on tomato plants, *Solanum* *lycopersicum* . Twospotted spider mites showed increased preference for and fecundity on TSWV-infected plants compared to mock-inoculated plants. Transcriptome profiles of TSWV-infected plants indicated significant up-regulation of salicylic acid (SA)-related genes, but no apparent down-regulation of jasmonic acid (JA)-related genes which could potentially confer induced resistance against TSM. This suggests that there was no antagonistic crosstalk between the signaling pathways to influence the interaction between TSWV and spider mites. In fact, SA- and JA-related genes were up-regulated when plants were challenged with both TSWV and the herbivore. TSWV infection resulted in down-regulation of cell wall-related genes and photosynthesis-associated genes, which may contribute to host plant susceptibility. There was a three-fold increase in total free amino acid content in virus-infected plants compared to mock-inoculated plants. Total free amino acid content is critical for arthropod nutrition and may, in part, explain the apparent positive indirect effect of TSWV on spider mites. Taken together, these data suggest that the mechanism(s) of increased host suitability of TSWV-infected plants to non-vector herbivores is complex and likely involves several plant biochemical processes.

## Introduction

Plants can influence interactions between arthropod herbivores and pathogens in numerous ways; positive, negative or neutral [1]. It is widely-accepted that the relationship between a vector and the pathogen it transmits is one of mutualism, and numerous studies have documented such effects. For instance, positive effects of plant viruses on vector fitness have been shown for aphids [2-5], whiteflies [6], and thrips [7-10]. Beneficial effects of viruses on insect vectors include increased host preference and increased fecundity, development, population growth and survival on infected plants compared to healthy plants. Although not as well-studied, there is accumulating evidence that plant viruses can also impact fitness of non-vector arthropods. For example, survival of Colorado potato beetle was higher on tomato plants infected with Tobacco mosaic virus (TMV) than on healthy plants [11]. Development of *Spodoptera* *exigua* caterpillars was greater on TMV-infected tomato plants than on control plants [12]. Pepper plants infected with Tomato spotted wilt virus (TSWV) harbored greater populations of a non-vector arthropod, the twospotted spider mite, *Tetranychusurticae* Koch (Acari: Tetranychidae) than uninfected plants [13]. On the other hand, TSWV infection reduced fecundity and longevity of whiteflies on TSWV-infected pepper plants [14]. The impact of virus infection of the host plant on non-vector arthropod fitness cannot be predicted at present.

Various ecological and molecular mechanisms underpin interactions involving plants, pathogens, and herbivores. Two primary mechanisms thought to underlie plant-mediated pathogen-herbivore interactions are activation of defense-related signaling pathways and induction of primary and secondary metabolites [1]. Plants have diverse and coordinated networks of innate defensive responses to protect themselves against attack from insects and pathogens [15,16]. In nature, these stresses can occur either individually or in combination, as in the case of insect vectors that transmit pathogen during feeding. Depending on the nature of the attacker, either salicylic acid (SA) or jasmonic acid (JA) defense pathways are activated. Induced responses to pathogen attack are predominantly regulated by the phytohormone, SA, which results in the production of pathogenesis-related (PR) proteins that contribute to pathogen resistance [16]. In response to insect attack, plants mainly activate defense pathways that are regulated by the phytohormone, JA, which acts as a signaling molecule for the production of a several metabolites such as protein inhibitors that contribute to herbivore resistance [15]. These signaling pathways interact with one another and often result in suppression of responses by the other, known as antagonistic crosstalk. For instance, a negative effect of the SA-pathway on the JA-mediated signaling has been reported for several plant-pathogen-systems [17-21]. Several insect herbivores utilize this antagonistic crosstalk to modify and/or avoid effective JA-related plant defenses. For example, Abe et al. [10] found that TSWV infection up-regulated SA-related gene expression in *Arabidopsis* that suppressed JA-regulated genes induced by the insect vector, western flower thrips. This resulted in increased feeding by and greater thrips populations on TSWV-infected plants. Non-vector arthropods may exploit antagonistic crosstalk between signaling pathways as well. For instance, Preston et al. [22], showed that *Tobacco mosaic virus* infection of wild tobacco suppressed JA-related resistance to the non-vector, tobacco hornworm, *Manduca sexta*. Hence, insect herbivores can benefit from feeding on virus-infected plants that have reduced herbivore resistance.

Pathogen and insect attack can also alter a plant’s primary metabolism, including amino acids, carbohydrates, sugars, and water content [1]. These changes occur as a result of trade-offs between primary and secondary metabolism, but they can also arise from disruptions in nutrient transport in the vascular tissues. Alterations in primary metabolism can significantly influence nutrients available to arthropods and therefore their survival. For example, virus-infected plants have greater total free amino acids, soluble carbohydrates and starch content, which are positively correlated with population growth of aphid vectors on these plants [23,24]. Furthermore, insect and pathogen attack also lead to changes in plant growth, architecture and morphology. The typical chlorosis or yellowing observed in virus-infected plants is thought to attract insect vectors [25]. And the curling of the apical meristem that occurs due to virus infection has been shown to not only provide shelter and protection to vectors from natural enemies but also against abiotic stresses [24].

TSWV belongs to the genus *Tospovirus* and is the only plant-infecting virus in the family *Bunyaviridae*. It has a genome consisting of three negative or ambisense RNA segments (S, M, L) [26]. TSWV infects hundreds of plant species worldwide, including several economically important crops, and TSWV epidemics in the past have caused significant economic losses [27]. The virus is transmitted exclusively by thrips, the most efficient of which is the western flower thrips, *Frankliniella* *occidentalis* (Pergande) [28]. The twospotted spider mite (TSM), *T* *. urticae* , is a highly polyphagous herbivore that feeds on more than 180 plant species [29]. Both *F* *. occidentalis* and TSM often co-invade the same plant hosts causing severe feeding damage resulting in reduced yield and quality [30]. As mentioned earlier TSWV is thought to have a positive effect on TSM [13], but there are no published reports on the mechanism(s) underlying the apparent positive effect of TSWV on TSM.

We sought to investigate ecological consequences and molecular mechanisms underlying the positive interaction between TSWV and the non-vector arthropod TSM on the plant host, tomato, *Solanum* *lycopersicum* L. We conducted replicated greenhouse experiments to characterize global transcript-level expression, phytohormone levels, and plant quality (total free amino acid content) of tomato systemically-infected with TSWV, or infested with TSM, or plants challenged with both TSWV and TSM. The current study corroborated the finding that TSWV infection of plants enhances fecundity of and preference by TSM. We focused our search of the tomato transcriptome for differential-expression of genes associated with phytohormone biosynthesis, signal transduction, transcription, and execution of defense to determine if there was evidence of crosstalk between SA and JA-dependent pathways that may explain enhanced host suitability for both arthropods. Our data revealed no apparent antagonistic crosstalk between SA- and JA-related genes; furthermore, SA- and JA-related genes were co-induced in plants challenged with both TSWV and TSM. There was strong evidence that TSWV infection enhanced levels of total free amino acids in infected plants that may, in part, explain increased numbers of TSM immatures on TSWV-infected plants.

## Results

### Effect of TSWV-infection of tomato on TSM fecundity and host preference

**Figure 1:**
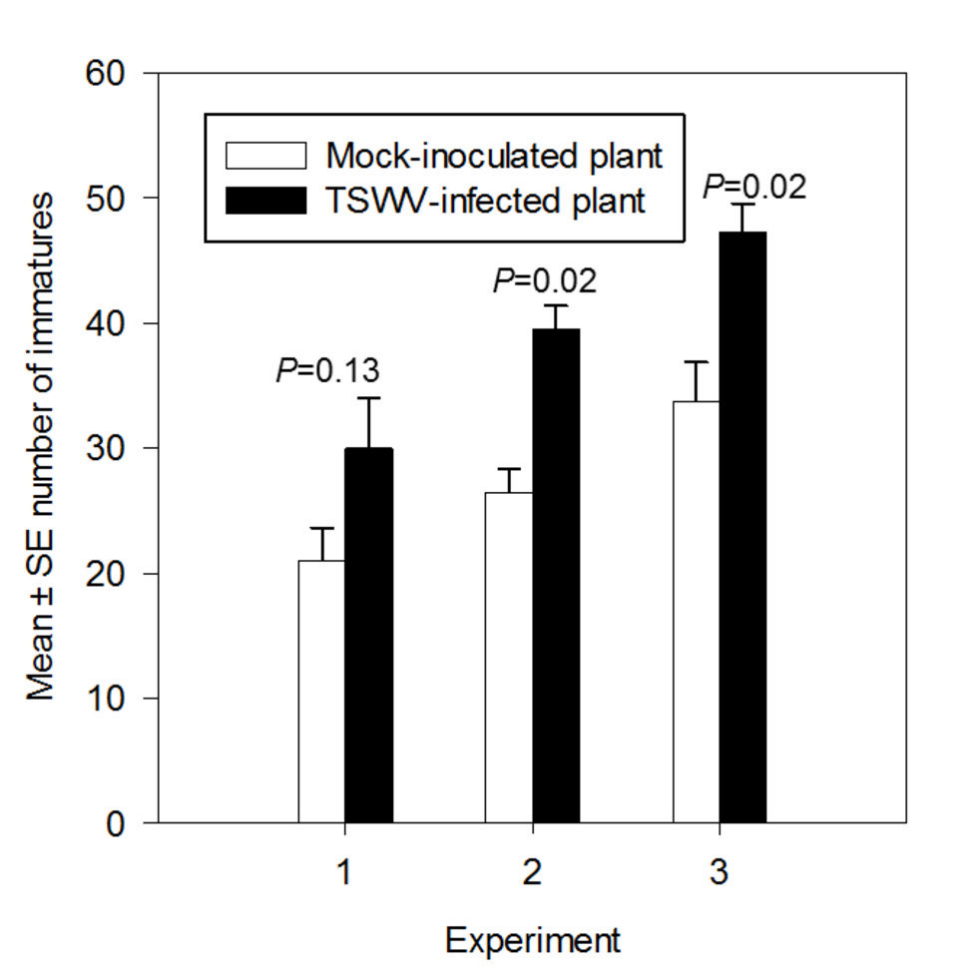
Effect of TSWV infection of tomato plants on fecundity of TSM. Number (mean ± standard deviation) of TSM offspring on tomato plants one-week after arthropod release in greenhouse experiments. Each bar represents the average of n=3-4 plants per experiment or biological replicate.

**Figure 2:**
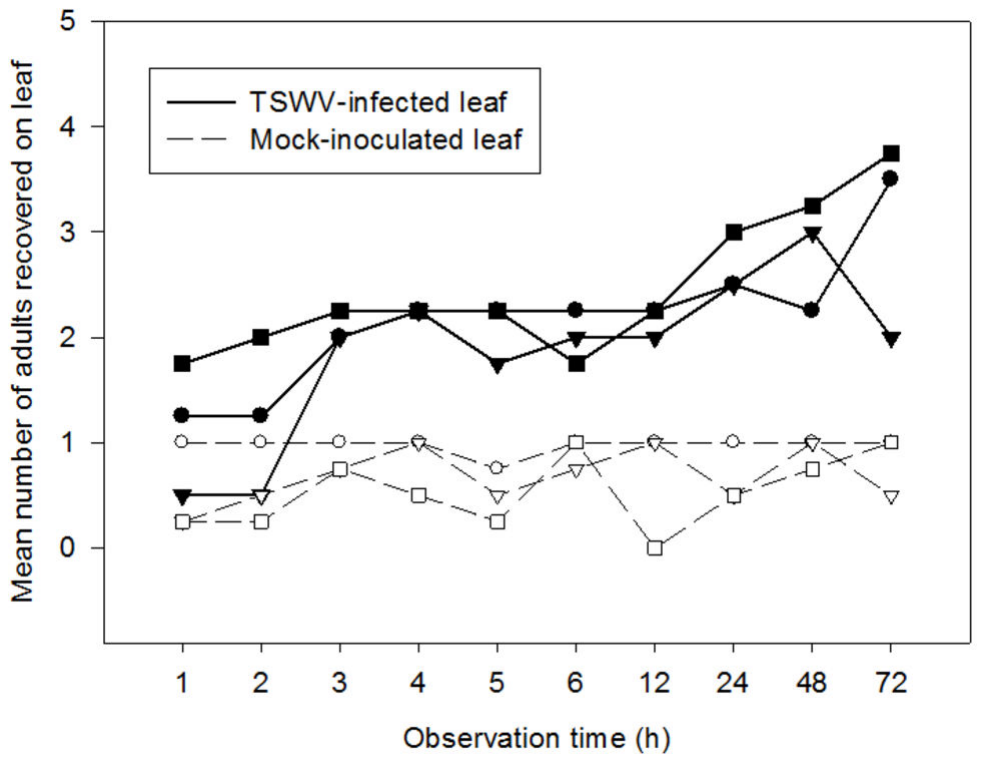
Effect of TSWV infection of tomato plants on host preference of TSM. Number of adult female TSM recovered on TSWV-infected and mock-inoculated leaflet in detached Petri dish leaflet assays. Leaflet pairs were obtained from TSWV-infected and mock-inoculated tomato plants from the corresponding greenhouse experiment or biological replicate. Lines connecting circles, triangles, and squares (open and solid) represent biological replicate (experiment) 1-3, respectively. Values at each time point represent the average of n=3-4 leaflets originating from a different experimental unit in the greenhouse experiment.

Virus-infected plants harbored 1.3 to 1.5 fold more TSM immatures than mock or healthy-leaf tissue-inoculated plants; in two of the three experiments these differences were significant (Figure 1). The average number of TSM immatures over the three experiments was 39.7 ± 2.6 (Mean ± SD) on TSWV-infected plants and 27.6 ± 2.1 on mock-inoculated plants. Petri dish choice tests showed that by three and four hours post release, there were significantly more TSM (*P* < 0.02) adults located on TSWV-infected leaflets than on mock-inoculated leaflets (Figure 2). This trend persisted over the course of the 72-hour experiment. There were no significant differences in the numbers of arthropods observed between leaflets in the no-choice test (*P* > 0.2, data not shown). Our results indicate that TSWV infection improves host plant suitability for the non-vector arthropod, positively affecting fecundity and host preference.

### Global gene expression profile

**Figure 3:**
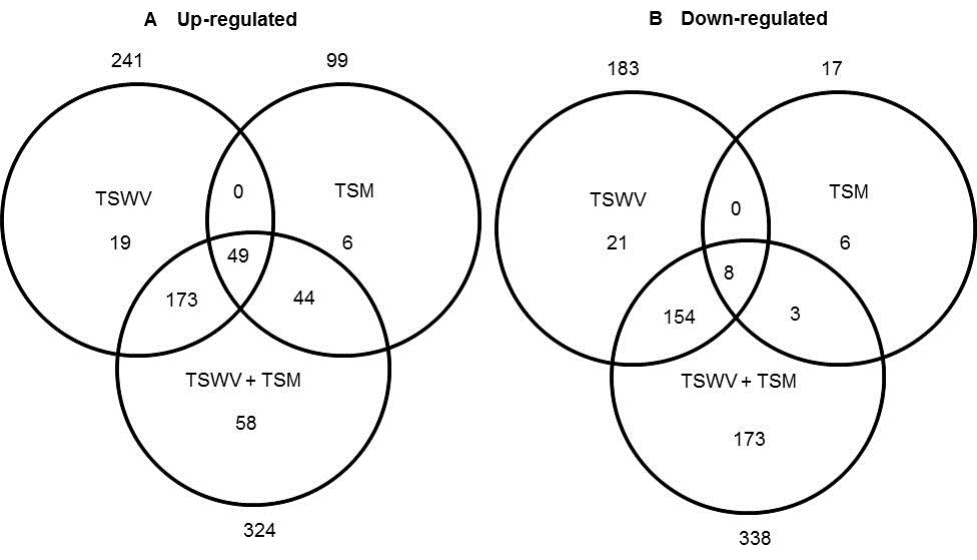
Venn diagrams depicting number of unique and shared differentially-expressed genes.

Of the 10,000 probe sets (i.e., 9,200 unique coding sequences) represented on the tomato GeneChip, 1,895 sequences were differentially-expressed in plants inoculated with TSWV, TSM, or TSWV+TSM compared to mock-inoculated plants (*P* < 0.05, regardless of magnitude of fold change) (Table S1). Of these sequences, 424, 116, and 662 genes were differentially-expressed at 2-fold in TSWV, TSM, and TSWV+TSM infected plants, respectively (Table S2, S3 and S4, respectively). Venn diagrams depicting the number of unique and shared genes that were differentially-expressed among treatments revealed several patterns (Figure 3). First, single attackers (TSWV or TSM alone) had fewer unique genes compared to dual attackers (TSWV +TSM). Second, the vast majority of genes that were shared between plant inoculated with TSWV, TSM, or TSWV+TSM were up-regulated (Figure 3A and B). These genes include SA-pathway related genes such as PR-5, subtilisin-like protease, endoglucanase, pathogenesis-related protein P2, and beta 1,3 glucanse. The eight genes that were down-regulated were largely unannotated except for one, myo-inositol-1-phosphate synthase. Third, the TSWV+TSM treatment resulted in a greater proportion of down-regulated genes that were unique to the treatment (Figure 3A and B).

 Data was obtained from differentially-expressed genes in tomato plants systemically-infected with TSWV and/or infested with TSM. Numbers outside of circles indicate the total number of differentially-expressed genes for a particular treatment.

The first three components (PC1, PC2, and PC3) of the PCA analysis explained 89% of the variation among the treatments. Analysis of variance on these scores revealed that expression patterns of the subset of genes associated with PC1 and PC2 were not significantly different among treatments (*P* = 0.27 and 0.41, respectively). However, expression patterns for genes associated with PC3 did reveal a significant overall difference among the treatments (*P* = 0.09), and this difference was attributed to the gene expression pattern in tomatoes infested by TSM alone compared to the combination treatment (TSWV+TSM). Genes associated with PC3 were largely protein metabolism genes (16% and 54%, positive and negative correlation, respectively). The second largest category in PC3 was response to stress (17% positive correlation).

### Gene ontologies of differentially-expressed tomato genes

**Figure 4:**
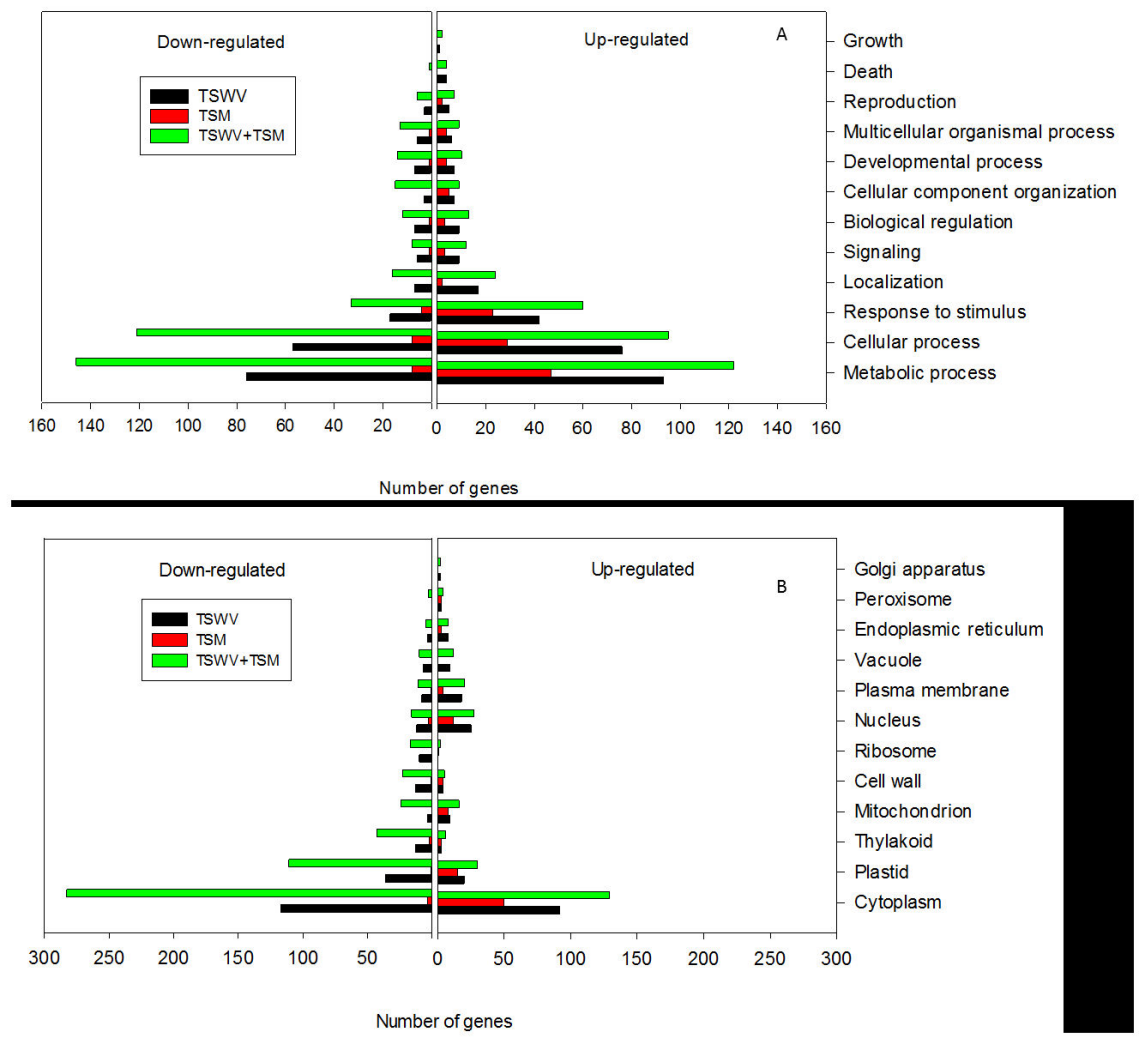
Gene ontology (GO) terms for differentially-expressed genes. Distribution of differentially-expressed genes in tomato plants systemically-infected with TSWV and/or infested with TSM. (A) Biological Process, (B) Cellular Component.

Differentially-expressed genes identified in the microarray analysis were functionally classified by Gene Ontology (GO) terms into 9 biological processes (level 2) (Figure 4A) and 12 cellular components (levels 5, 6 and 7) (Figure 4B) with relevance to primary and secondary metabolism. Overall, a greater proportion of genes within these broad categories responded to TSWV infection than to TSM infestation (Figure 4A and B). The GO biological process most represented by the differential expression was response to metabolic process. Within this category protein metabolism genes were most abundant, which was also reflected in the PCA (Figure 4A). Genes differentially-expressed in response to stimulus including abiotic (salt, light, water, nutrient, and temperature) and biotic stimulus (virus, bacteria, insect and wounding) was the third highest category. Less than 10% of the stress-related genes were specific to phytohormone-related defense signaling pathways. Genes associated with cell death, although few in number, were positively and primarily regulated by virus infection. There was a greater proportion of down-regulated genes in each of the cellular component category compared to up-regulated genes (Figure 4B). The GO cellular component overrepresented in differentially-expressed genes was the cytoplasm. The next largest category was the plastid followed by the thylakoid. Both cellular categories were down-regulated by TSWV alone and TSWV+TSM. A similar trend was observed for cell wall-related genes.

### Expression of genes associated with defense signaling pathways

The vast majority of the differentially-expressed defense-related genes (i.e., genes involved in SA, JA, and ethylene pathways) were those encoding the proteins that execute defense or limit damage (i.e., glucanase, chitinases, proteases, proteinase inhibitors, polyphenol oxidase) caused by pathogen infection or feeding and other signatures of transcriptional response downstream of SA and JA biosynthesis, for example PR proteins (Table 1). TSWV infection alone significantly up-regulated signature SA-responsive genes compared to mock-inoculated plants. Other TSWV- responsive defense genes included heat shock proteins, HSP70 and HSP17.6, and genes involved in detoxification of reactive oxygen species (ROS) such as superoxide dismutase, catalase, and peroxidase (Table 1). Key signaling and transcription factors that play roles in SA and JA signaling pathways (MAP kinases and WRKYs) were consistently and positively up-regulated in leaves of TSWV-infected tomato, and in most cases this up-regulation occurred regardless of the presence of TSM. There was no major effect of TSWV on the expression of JA-associated genes compared to mock-inoculated plants. However, there was a consistent but not significant trend of down-regulation of JA genes (9 out of 13) in the former compared to the latter. The presence of TSM alone on leaves resulted in up-regulation of 33% of SA-responsive genes, and TSM resulted in up-regulation of 85% of the differentially-expressed JA-related genes (Table 1). Also, evidence of crosstalk was not apparent in TSWV-infected leaves that were challenged with TSM; in fact, all SA and JA-associated genes in the TSWV+TSM treatment were up-regulated, indicating the major effects of the herbivory on these genes even in the presence of the virus in the leaf tissue.

**Table 1:** Microarray hybridization intensities of differentially-expressed genes involved in plant defense responses.

**Gene Title**	**Probe set ID**	**P-value**	**MOCK**		**TSWV**		**TSM**		**TSWV + TSM**	
**SA-associated genes**										
Beta 1,3, glucanase	Les. 3673.1.S1_at	<0.001	7.44	b	12.45	a	8.44	b	12.27	a
Non-inducible immunity 1	Les. 5940.1.S1_at	0.005	7.94	b	8.93	a	8.07	b	9.23	a
Pathogenesis-related protein-5	Les. 3683.1.S1_at	<0.0001	6.84	c	12.56	a	9.97	b	12.47	a
Pathogenesis-related protein-2	Les. 4460.1.S1_at	0.01	9.48	c	12.64	ab	10.77	bc	13.12	a
Pathogenesis-related protein-2	Les. 3154.1.A1_at	0.03	12.28	a	11.6	b	12.33	a	11.44	b
Pathogenesis-related protein	Les. 3408.1.S1_at	0.031	11.81	b	13.62	a	13.44	a	13.56	a
Subtilisin-like protease	Les. 3635.1.S1_at	0.0002	9.8	c	12.36	a	11.02	b	12.25	a
Subtilisin-like endoprotease	Les. 3648.1.S1_at	0.03	9.89	a	9.22	ab	9.91	a	8.93	b
Tobacco stress-induced 1	Les. 4496.1.S1_at	0.02	11.22	b	12.99	a	12.2	ab	13.25	a
**JA-associated genes**										
Allene oxide synthase	Les. 13.1.S1_at	0.02	7.31	ab	5.73	b	8.32	a	8.28	a
Cathepsin D inhibitor protein	Les. 3740.1.S1_at	0.01	9.66	b	7.75	b	13.72	a	13.73	a
Cathepsin D inhibitor protein	Les. 3035.1.A1_at	0.004	13.29	b	12.57	b	14.12	a	14.06	a
Cysteine protease inhibitor	Les. 4820.1.S1_x_at	0.01	4.74	b	4.66	b	9.16	a	9.22	a
Leucine aminopeptidase	Les. 84.1.S1_at	0.01	10.69	b	10.25	b	13.23	a	13.61	a
Metallocarboxypeptidase inhibitor	Les. 3974.1.A1_at	0.03	11.07	b	11.16	b	13.87	a	13.96	a
12-oxophytodienoate reductase	Les. 22.1.S1_at	0.02	11.41	b	12.28	a	11.59	b	12.3	a
Polyphenol oxidase A	Les. 4528.1.A1_at	0.02	4.24	bc	4.05	c	4.44	ab	4.71	a
Prosystemin	Les. 2121.1.A1_at	0.01	8.11	c	8.36	bc	9.07	ab	9.23	a
Threonine deaminase	Les. 4488.1.S1_at	0.02	12.06	b	11.58	b	13.81	a	13.82	a
Wound-induced proteinase inhibitor II	Les. 1675.1.S1_at	0.01	10.81	b	8.73	b	14.3	a	14.07	a
Wound-induced proteinase inhibitor II prepeptide	Les. 1675.1.S2_at	0.05	11.39	ab	11.73	a	11.06	b	11.61	a
Wound-inducible carboxypeptidase	Les. 3515.1.S1_at	0.04	11.18	bc	10.87	c	12.13	ab	12.35	a
**Et-associated genes**										
Ethylene receptor homolog 1	Les. 3490.1.S1_at	0.002	9.53	b	9.91	a	9.55	b	10.05	a
Ethylene response factor 5	Les. 4531.1.S1_at	0.01	9.18	c	9.52	a	9.25	bc	9.41	ab
Ethylene-insensitive 3-like 1 protein	Les. 3472.1.S1_at	0.04	11.12	b	11.46	ab	11.38	ab	11.75	a
Ethylene-responsive transcriptional coactivator	Les. 3551.1.S1_at	0.03	7.98	ab	10.47	a	6.88	b	10.55	a
Ethylene responsive protein 33	Les. 126.1.S1_at	0.001	9.92	b	11.72	a	10.02	b	11.51	a
Ethylene-insensitive 3-like 3 protein	Les. 3470.1.S1_at	0.03	10.95	b	11.5	a	10.89	b	11.64	a
**Transcription factors**										
Mitogen activated protein kinase kinase	Les. 2855.1.S1_at	0.02	10.11	b	10.99	a	10.46	ab	11.01	a
Mitogen activated protein kinase 4	Les. 5954.1.S1_at	0.02	11.01	b	11.87	a	10.91	b	12.06	a
Mitogen activated protein kinase 2	Les. 5948.1.S1_at	0.04	9.87	b	10.34	a	9.89	b	10.43	a
Myb-related transcription factor	Les. 3676.1.S1_at	0.001	4.99	b	6.65	a	5.68	b	7.41	a
WRKY transcription factor IId-3	Les. 3962.1.A1_at	0.05	7.34	c	8.2	a	7.39	bc	8.06	ab
WRKY transcription factor IId-4	Les. 546.1.A1_at	0.01	7.84	b	9.53	a	8.66	ab	9.73	a
WRKY transcription factor IId-6	Les. 3961.1.S1_at	0.01	9.29	b	10.08	a	9.11	b	9.97	a
**Other defenses**										
Chitinase	Les. 122.1.S1_at	0.0002	11	c	13.82	a	12.85	b	13.8	a
Carbonic anhydrase	Les. 796.1.A1_at	0.05	6.45	ab	5.42	b	9.03	a	9.48	a
Cytosolic class II small heat shock protein HCT2	Les. 3578.1.S1_at	0.02	6.28	bc	8.08	a	5.68	c	7.46	ab
Class II small heat shock protein Le-HSP17.6	Les. 3581.1.S1_at	0.05	6.52	ab	8.52	a	4.94	b	7.35	ab
Ethylene-responsive heat shock protein cognate 70	Les. 3550.1.S1_at	0.001	8.11	b	10.08	a	7.29	b	9.35	a
Heat shock induced transcript 2	Les. 4456.1.S1_at	0.003	10.03	b	10.53	a	10.12	b	10.79	a
NADPH oxidase	Les. 26.1.S1_at	0.02	6.21	b	7.29	a	5.84	b	6.63	ab
Superoxide dismutase	Les. 167.1.S1_at	0.01	12.42	a	10.52	b	12.37	a	11.05	b
RNA-directed RNA polymerase	Les. 61.1.S1_at	0.01	9.1	b	10.44	a	9.4	b	10.63	a

Average hybridization intensities in plants systemically-infected with TSWV and/or infested with TSM. Values represent mean of three biological replicates. Mean values followed by a different letter represents significant differences between treatments (*P* < 0.05).

### Reverse transcription-quantitative PCR (RT-qPCR) validation of microarray hybridization data and virus titer in tomato leaves

A subset of SA and JA genes that was determined to be differentially-expressed by TSWV, TSM and TSWV+TSM in the microarray hybridization experiment was further analyzed by reverse transcription-quantitative PCR (RT-qPCR) to confirm the direction of expression (Table 2). We included RDR1, a key siRNA-pathway gene involved in the amplification of virus derived siRNAs that target viral dsRNAs for degradation, to examine antiviral defense. For the most part, the average relative expression ratios of SA and JA genes in response to virus infection with and without TSM mirrored the direction (positive or negative) of expression for the microarray analyses (Table 2). Furthermore, pairwise comparisons of treatment averages obtained for the microarray and real-time RT-qPCR analyses revealed similar patterns among treatments. RDR1 expression was up-regulated in TSWV-infected as compared to TSM fed tissue (*P* < 0.05) (Table 2).

**Table 2:** Reverse transcription quantitative-PCR (RT-qPCR) validation of differential genes.

		**Log_2_(Fold change intensity**)			**Log_2_(Relative expression ratio**)		
**Gene title**	**Probe set ID**	**TSWV**	**TSM**	**TSWV +TSM**	**TSWV**	**TSM**	**TSWV +TSM**
**SA pathway**							
Beta 1,3, glucanase (BGL2)	Les. 3673.1.S1_at	5.1a	1.0b	4.8a	6.0a	1.8b	5.7a
Non-immunity 1 (NIM1)	Les. 5940.1.S1_at	1.0a	0.1b	1.3a	2.3a	0.5a	2.4a
**JA pathway**							
Allene oxide synthase (AOS)	Les. 13.1.S1_at	-1.6b	1.0a	1.0a	-0.2a	1.5a	2.1a
Cathepsin D inhibitor protein (CI)	Les. 3740.1.S1_at	-1.9a	4.7a	4.7a	-1.5b	7.8a	9.0a
**RNAi pathway**							
RNA-directed RNA polymerase 1 (RDR1)	Les. 61.1.S1_at	1.3a	0.3b	1.5a	2.2a	1.3b	3.2a

Relative transcript abundance in tomato plants systemically-infected with TSWV and/or infested with TSM. Values represent mean of three biological replicates. Mean values followed by a different letter represents significant differences (*P* < 0.05) between treatments for a particular gene and type of measurement.

The normalized abundance of TSWV nucleocapsid (N) RNA compared to EF1 was also determined to estimate virus titer in leaf tissue using primers tested previously [31]. Virus titers in tomato leaflets used in the microarray and RT-qPCR expression assays were determined and compared to relative expression ratios of the subset of four SA and JA-associated defense genes. The average Ct values obtained for real-time RT-qPCR amplification of TSWV N ranged from 11.6-23.8, with the majority of values closer to 11.6. The Log_2_-transformed normalized abundance (NA) of TSWV N to EF1 (i.e., virus titer) ranged from 9.1 to 16.6. The presence of TSM on infected leaves had no measurable effect (NA_virus_ = 12.6; NA_virus+TSM_ = 12.1; *P* = 0.85) on TSWV titer in these leaves and there were no significant correlations (*P* > 0.49) between virus titer and RERs of any of the four defense genes further analyzed by RT-qPCR.

### Phytohormone levels in leaf tissue

TSWV infection alone significantly increased SA levels, but there was no difference in JA levels compared to mock-inoculated plants (Table 3). This virus effect mirrored the expression of SA and JA genes in TSWV-infected plant in the microarray analyses (Table 1). TSM infestation significantly increased JA content in the leaf. There was a significant increase in SA level and a moderate increase in JA level in the dual treatment (Table 3). There were no apparent differences among treatments with regards to JA-IL or OPDA at time of sampling.

**Table 3:** Phytohormone content in tomato plants systemically-infected with TSWV and/or infected with TSM.

**Phytohormone**	**MOCK**	**TSWV**	**TSM**	**TSWV+TSM**
Salicylic acid	131.53 + 85.34b	4399.75 + 407.97a	555.22 ± 95.99b	8288.66 + 744.48a
Jasmonic acid	1.24 + 0.11b	2.22 + 1.50b	7.61 + 4.56a	3.46 + 1.42ab
JA-Isoleucine	4.59 + 2.24a	1.98 + 0.99a	13.98 + 4.88a	7.37 + 3.48a
OPDA (12-oxo-phytodienoic acid)	25.71 + 6.87a	34.23 + 9.01a	155.52 + 45.78a	233.18 + 44.54a

Values are expressed as ng analyte g fresh weight^- 1^. Values represent mean + standard deviation of three biological replicates. Mean values followed by a different letter represents significant differences between treatments (*P* < 0.05).

### Total free amino acid content

**Figure 5:**
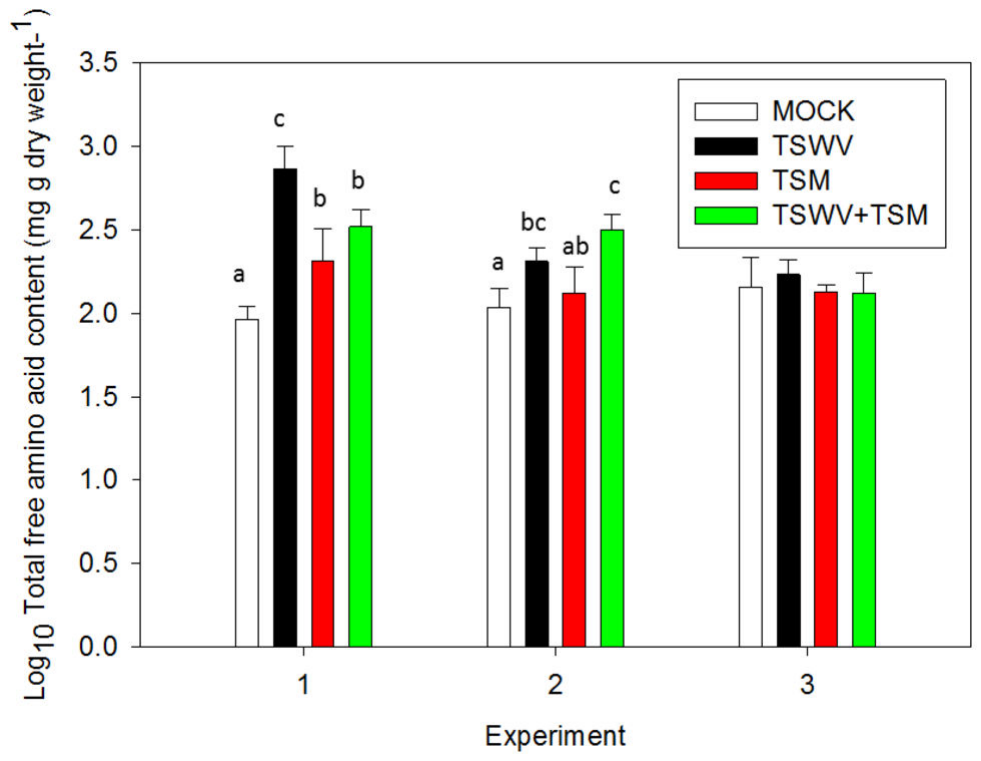
Total free amino acid content in tomato systemically-infected with TSWV and/or infested with TSM.

TSWV infection with or without TSM was associated with greater free amino acids in two of three replicate experiments (experiment 1 and 2; Figure 5). These experiments correspond with fecundity experiment (2 and 3) (Figure 1). In experiment 3 of the amino acid analyses, there was no significant difference in free amino acids and corresponding fecundity of TSM on mock- and virus-infected plants (Data not shown, *P* =0.19) (Figure 5). Overall, TSWV infection alone increased total free amino acid content 3.2 times compared to mock-inoculated plants, and TSWV+TSM increased amino acid content 2.2 times. The effect of TSM on free amino acid content was significant in only 1 of the 3 the replicates, but there was an overall increase in amino acid content by 1.4 times compared to mock-inoculated plants.

 Each bar represents the mean ± standard deviation of n=4 plants per biological replicate. Bars with the same letter are not significantly different at *P* < 0.05. There were no significant differences among treatments in experiment 3.

## Discussion

We investigated plant-mediated interactions between a plant virus and a non-vector arthropod herbivore on tomato at several biological levels: ecological (host suitability measured as herbivore fecundity and host preference); physiological (total free amino acid and phytohormone content); and transcriptional (global gene expression profiles using tomato oligonucleotide microarrays). Our results support the hypothesis that plants systemically-infected with TSWV are more suitable for TSM, a non-vector arthropod, than are uninfected plants as a result of changes in the quality of plant tissues as food for TSM and to a limited extent by plant defensive response to TSM that were precipitated by TSWV.

Specifically, TSWV infection of tomato plants increased fecundity of TSM by 30% compared to mock-inoculated plants (Figure 1). A similar result was reported for TSM in another solanaceous crop species, *Capsicum annuum* (pepper) [13]. The authors showed that the number of TSM offspring was greater on mechanically-inoculated TSWV plants compared to mock-inoculated plants; no other life-history parameters were affected by virus infection. In addition, in Petri dish choice assays, TSWV infection of tomato caused TSM to aggregate on these leaflets in greater proportion than on mock-inoculated leaflets (Figure 2). One reason for the attraction of TSM to TSWV-infected plants may be the typical yellow color of these leaflets. For instance, TSWV-infected lettuce plants were more attractive for thrips vectors compared to healthy plants because of the yellow color of the infected plants [25]. It is also possible that TSWV infection releases volatile emissions that are attractive to feeding herbivores. Volatile emissions from other plant viruses (*Potato leafroll virus*) have been shown to attract aphid vectors to these leaflets compared to leaflets from uninfected plants [4]. Taken together aggregation and increased reproduction would lead to higher TSM population levels on TSWV-infected plants.

Plant mechanisms that enhance host plant suitability of virus-infected plants for arthropod herbivores are complex; our data suggest that TSWV infection may influence both plant nutritional quality and plant ability to mount defenses upon attack by virus and herbivore. We found that TSWV infection alone or in combination with TSM increased total free amino acid content in virus-infected plants (Figure 5). A previous study also reported that TSWV-infected plants have greater amounts of total free amino acid by 150-180% compared to healthy plants [32]. Increase in total free amino acids in virus-infected plants has been shown to be positively correlated with fecundity of aphid vectors [24]. There was an overabundance of genes involved in protein metabolism in the current study, which may explain the increased total free amino acid content in TSWV-infected plants, but the mechanism underlying increased amino acid content due to virus-infection is not well-understood. Expression profiles of shoots and roots of tomato infected with TSWV showed that amino acid metabolism was repressed in tomato shoots, but no analysis was performed on the leaves [33].

Our study showed that TSWV infection caused significant increase in SA-related gene expression including PR-1, PR-2 (β-1,3 glucanase), genes associated with redox status such as superoxide dismutase and glutathione S-transferases (GST), and other genes that are co-induced with the PR genes such as HSP. In addition, we detected up-regulation of NIM1 (NPR1), the regulatory protein that plays a vital role in modulation of SA and JA signaling transcription factors such as WRKY genes, AP2 and MYB genes, and MAPK4 (Table 1).

There was no apparent effect of TSWV infection on the JA signaling pathway genes compared to mock-inoculated plants (Table 1). Phytohormone analysis also revealed increased SA content, but no difference in JA levels in TSWV-infected plants (Table 3). There was a non-significant trend for lower average JA-related gene expression in 69% of the genes in TSWV-infected plants in the microarray analysis. Interestingly, RT-qPCR analysis indicated that Cathepsin D inhibitor protein was significantly down-regulated in TSWV-infected tissue (Table 2). These genes are predominantly proteinase inhibitors that serve as markers for the JA-signaling pathway and have an anti-feeding effect on herbivores. SA-mediated suppression of JA-responsive gene expression is thought to mainly occur downstream of the JA biosynthesis pathway [34]. This may explain the down-regulation of JA-responsive genes such as Cathepsin inhibitor genes, and the lack of down-regulation in JA biosynthesis genes such as AOS in TSWV-infected plants analyzed in RT-qPCR analysis (Table 2). Moreover, AOS mRNA accumulation is more transient than PI mRNAs in tomato [35]. It is also possible that SA and JA signal antagonism may be specific to particular genes such as proteinase inhibitors in tomato. Abe and co-workers [10] demonstrated that TSWV-infection in *Arabidopsis* resulted in down-regulation JA-related genes LOX2 and VSP2, which presumably led to suppression of anti-herbivore responses, thereby increasing fitness of its vector, western flower thrips (the vector) on TSWV-infected plants. However, those authors analyzed expression of SA- and JA-related marker genes 7d and 14d post-TSWV infection, whereas in our study gene expression was measured 21d post-virus infection. During insect and pathogen attack, the interactions between SA and JA signaling pathways are highly dynamic [36], but little is known about time-dependent changes in induced defense responses. Koornneef et al. [36] showed that JA transcripts were down-regulated when SA was exogenously applied 24h after insect infestation or JA induction. However, when SA was applied more than 30h prior to insect infestation, there was no down-regulation of JA genes. In the present study, virus inoculation was performed two weeks prior to TSM infestation, which may be one reason for the absence of antagonistic crosstalk in TSWV-infected tissues. Collectively, these reports suggest that SA-related suppression of JA-related genes may influence host plant resistance against vector and non-vector arthropods.

We found a greater proportion of differentially-expressed tomato genes in response to TSM feeding when compared to the findings of Kant et al. [37]. These differences may reflect variation in methodology and timing of leaf tissue sampling used in the two studies. Kant et al. [37] found that TSM feeding induced expression of JA-responsive genes, particularly wound-induced proteinase inhibitor, as early as 1 d after feeding and expression remained high after 4d. In contrast, expression of JA biosynthesis enzymes, LOX and AOS only showed a transient and gradual increase by 2 d in response to TSM feeding [38]. Expression of SA-regulated genes such as various acidic chitinases and the pathogenesis-related TSI-1 (tomato stress induced-1) were high at 4d post-TSM feeding. In our study, TSM feeding alone resulted in significant up-regulation of 9 out of 13 of the JA-related genes and 3 out of 9 of SA-responsive genes, and TSM feeding does not seem to affect expression of ET-responsive genes (Table 1). In summary, induced defense responses to TSM feeding is regulated mainly by JA pathway and to a lesser extent by the SA pathway.

The combination of TSWV and TSM treatments mirrored gene expression patterns of TSWV infection alone, which suggests that TSWV infection has a significant effect on plant physiology relative to TSM (Figure 4A and B). Interestingly, the net effect of TSWV infection and TSM infestation resulted in a positive effect on SA- and JA-responsive genes (Table 1). Although, the interaction between SA- and JA-signaling pathways is mainly thought to be antagonistic [39], there are reports of synergistic interaction. Schnek and co-workers [40] showed that application of SA and methyl jasmonate results in a well-coordinated defense response that involves both co-induction and co-repression of genes, with greater number of co-induced genes (55) than co-repressed genes (28). Co-treatment of SA and JA elicitors at a low concentration resulted in synergistic effect of defense response genes, whereas prolonged treatment or at higher concentrations resulted in an antagonistic effect [41]. Our findings suggest that in response to infection by a single attacker, namely TSWV alone, plants induce SA-mediated defense responses while concomitantly reducing JA-mediated responses. However, when faced with TSWV infection and TSM simultaneously, plants modify their defenses by the relative activation of both SA- and JA-mediated defenses, leading to optimal defense against both the attackers.

In addition to induced defenses, pathogen and herbivore attack can alter constitutive plant traits such as leaf thickness, toughness and color that can potentially influence outcomes of plant-pathogen-vector interactions [1]. In the current study, TSWV-infection alone and in combination with TSM resulted in the down-regulation of cell wall genes such as xyloglucan endotransglucosylase-hydrolase, pectinesterase, expansion, extension, and cell wall invertase (Figure 4B). It is possible that suppression of cell wall genes renders the plant more susceptible to the penetration of mouthparts of feeding herbivores. Several chloroplast-related genes associated with the plastid and thylakoid, including chlorophyll a/b-binding protein, ribulose bisphosphate carboxylase, and photosystem II proteins, were also down-regulated in TSWV-infected plants compared to mock-inoculated plants (Figure 4B). These changes may be associated with the development of chlorotic or yellowing symptoms of virus-infected plants, which may explain the preference of arthropods to the yellow color associated with virus-infected plants [24].

## Conclusions

We found strong evidence that TSWV infection increased the total free amino acid content in tomato plants, which may, in part, explain the increased fecundity of TSM on these plants. We did not detect an antagonistic effect of TSWV-induced SA-defenses on JA-mediated defenses, which could potentially induce resistance against TSM. Further experimentation utilizing mutants of the SA- and JA-signaling pathways would be useful to explore this result. There is also a need to determine gene expression profiles during early versus late stages of infection or disease development. In plants challenged by both TSWV and TSM, there was an overall co-induction of SA- and JA-mediated responses. This suggests that plants have evolved sophisticated and well-coordinated network of responses to combat multiple attackers at once. The ability of pathogens and herbivores to successfully colonize plants suggests that they have evolved to counter plant defenses. One such adaptation is the modulation of the plant’s own induced defense response by pathogens and herbivores for their own benefit. For instance, salivary secretions of piercing-sucking insects prevent plant cell wall damage and phloem leakage, which could trigger induced defense responses. Recently, it was demonstrated that an effector from Aster yellows *Phytoplasma* , SAP11 inhibits expression of LOX2 resulting in increased performance of the vector on *Phytoplasma* -infected plants [42]. There is also indication that virus pathogenicity proteins, betaC1 of *Tomato yellow leaf curl China virus* [43] and 2b of *Cucumber mosaic virus* [44] inhibit JA-responsive genes, which can influence virus transmission by vectors. The beneficial effect of the viruses on non-vector arthropods is surprising because the virus has no apparent benefit from non-vectors. It is possible that non-vector arthropods exploit changes in plant physiology due to virus infection such as repression of anti-herbivore defense and increased amino acid content similar to vectors by attacking virus-infected plants. Future studies may be aimed at determining the identity of specific TSWV and herbivore factors that can modulate plant response to enhance herbivore performance on virus-infected plants.

## Methods

### Virus and TSM source

TSWV (isolate TSWV-MT2) was maintained by mechanical inoculations on tomato ( *Solanum* *lycopersicum* cv Moneymaker) plants in the greenhouse. Twospotted spider mites (TSM) were reared on lima bean (*Phaseolus lunatus* cv. ‘Sieva’) plants in a rearing room at 24 + 1°C and a photoperiod of 16: 8 h (light:dark).

### Plant source

Tomato plants were grown in 15.2-cm pots filled with Metro mix® potting soil and each pot was housed in a thrips-proof-screened (No Thrips Insect Screen, BioQuip Products, Rancho Dominguez, CA) cage in a greenhouse. Temperatures ranged from 23-25°C and the photoperiod was 16: 8 h (L: D). Plants were fertilized once a week with Miracle Gro®-Water Soluble All Purpose Plant Food (24-8-16) NPK.

Three weeks after germination, half of the plants were mechanically-inoculated with TSWV and the other half were mock-inoculated with buffer and healthy plant tissue. The virus inoculum was prepared by grinding two to three symptomatic young tomato leaves in ice-cold 5-10 ml of inoculation buffer (10 mM sodium sulfite and 5% wt/vol celite) using a pre-chilled mortar and pestle. Inoculum was applied and dragged lightly with a cotton swab over the surface of all fully-expanded leaves on the plant. Approximately two weeks after inoculation, plants were visually inspected for TSWV symptoms (i.e., stunting and deformation, chlorotic ring spots, mosaic patterns, and leaf-bronzing), and the most uniform group of symptomatic plants were chosen for each experiment.

### Greenhouse experimental design and structure

The greenhouse experiment consisted of four treatments: 1) TSWV infection alone, 2) TSM infestation alone, 3) TSWV and TSM, and 4) mock-inoculated or uninfected controls. Each treatment was replicated three or four times (i.e., three or four plants per treatment) in a randomized complete block design with one plant per treatment per block. The experiment was conducted three times (i.e., biological replication).

Individual 5-week-old infected and uninfected control plants were moved to single-plant cages constructed from 19-liter cardboard ice cream buckets (38 cm tall × 26 cm in diameter) with four 14 × 27 cm apertures cut into the side walls. The four apertures were covered with No-Thrips Insect Screen™ (GreenTek, Inc., Edgerton, WI, USA) and sealed with silicone. The top of the container was covered by thrips-proof-screen secured by rubber bands. These single-plant cages prevented cross-contamination between treatments. Fifteen adult female TSM (7-days old) were transferred using a camel-hair brush onto the adaxial surface of each of the terminal trifoliate tomato leaflets of the 4^th^ youngest leaf (i.e., 45 mites per leaf per plant).

To assess plant quality as a food resource, TSM fecundity was measured at seven days post-release by counting the number of immatures deposited on leaf surfaces. For each biological replicate, a 2-sample Mann-Whitney rank test was performed using Minitab v.14 (Minitab, Inc., State College, PA) to determine if the median count of offspring obtained from TSWV-infected plants was similar to that obtained from uninfected plants.

To determine host preference between TSWV- and mock-infected plants, a detached-leaflet assay was designed to allow TSM to choose between paired leaflets. Tests were performed in 15-cm-diameter Petri-dishes containing 15 ml of 1.5% water agar to prevent desiccation of leaflets while allowing movement of the TSM across the surface. The lids of each dish were fitted with thrips-proof screen to ventilate the system. The adaxial surface of each leaflet was placed in contact with the agar surface. Preference was measured by number of TSM that aggregated on a given leaflet. For choice tests, TSM were placed in Petri dishes with a leaflet from a TSWV-infected plant and a leaflet from a mock-infected plant. For the no-choice tests (controls), TSM were placed in petri dishes that had two mock-inoculated leaflets or two TSWV-infected leaflets. Same-age leaflets were obtained from mock-inoculated plants and plants with TSWV infection alone at the termination of each greenhouse experiment (i.e., 6-week-old plants). In each test, ten adult female TSM were placed with a small paint brush in the center of the Petri dish, equidistant from each leaflet, and lids were sealed with parafilm. The assay was conducted under laboratory conditions with a 16: 8 h (L:D) photoperiod and ambient temperatures of 24-25°C. The number of TSM present on each of the paired leaflets were counted every hour for the first six hours, and then at 12, 24, 36, 48, and 72 h after the release. For each biological replicate, 1-sample Wilcoxon sign rank tests were performed using Minitab to determine if the median paired difference in accumulation of TSM on virus-infected vs. mock-inoculated tissue, mock-inoculated vs. mock-inoculated tissue, and virus-infected vs. virus-infected tissue was significantly greater than zero at each time-point.

### Leaf tissue sampling for molecular and total free amino acid analyses

One week after TSM infestation, two same-age leaflets (one from each side of leaf rachis) were harvested from the 7^th^ youngest cohort of leaves, from each of the four treatments (TSWV infection alone, TSM infestation alone, TSWV and TSM, and mock-inoculated or uninfected controls). In the TSM and TSWV+TSM treatment this leaf was originally exposed to mites in the 4^th^ leaf, i.e., the origin of placement resulting in the 7^th^ leaf at time of sampling. Leaflet samples were flash-frozen in liquid nitrogen and stored at -80°C. One leaflet was processed for global gene expression analysis (i.e., microarray hybridizations) and the other was processed to determine phytohormone contents. To obtain enough leaf tissue (approximately 5 g) required for determination of total free amino acid content, a third leaflet immediately basal to the leaflets chosen for microarray and phytohormone analyses was harvested and freeze-dried. The leaf tissues for microarray and phytohormone analyses were harvested from the corresponding plant used to assess fecundity and host preference from each of the three biological replicates (experiment 1, 2 and 3). The total free amino acid analysis was not performed on leaf tissues from experiment 1; hence, a separate experiment (experiment 4) was conducted to specifically obtain leaf tissues for amino acid analysis. Overall, there were three biological replicates for microarray and total free amino acid analyses.

### Global gene expression in leaf tissue

Total RNA was isolated from frozen leaflet samples (100-200 mg of tissue) using the Qiagen RNeasy Plant Mini Kit (Qiagen, Valencia, CA, U.S.A.) following manufacturer’s protocol. RNA concentrations were determined by NanoDrop spectrophotometer (NanoDrop Technologies, Wilmington, DE, USA) and quality assessed with the 2100 Bioanalyzer using Nanochip technology (Agilent Technologies, Inc., Palo Alto, CA, USA). Total RNA was pooled for each treatment (3-4 experimental replicates, i.e., plants) within a biological replication. There were three biological replicates or microarray per treatment.

Pooled RNA samples were sent to the Kansas State University Integrated Genomics Center for cRNA synthesis, labeling, and hybridization to Affymetrix Tomato Genome Arrays (GeneChip) following Affymetrix’s standard protocol. Each GeneChip contained more than 10,000 probe sets for over 9,200 genes with each gene being represented by at least one probe set containing 25-mer oligonucleotides. Signal intensities of scanned microarrays for each of the three biological replicate were generated with Gene Chip Operating System, GCOS (Affymetrix Inc.). Global scaling was applied for each GeneChip to adjust the Target Intensity (TGT) Value to an arbitrary target of 500 so that hybridization intensity of all chips was equivalent. In addition, expressed genes were identified by GCOS, using a detection algorithm and assigned a present, marginal, or absent call for genes represented by each probe set on the array (GeneChip Expression Analysis Technical Manual). Microarray data files (.CEL) were analyzed using GeneSpring 10.1 (Silicon Genetics, Redwood, CA, USA) and normalized using RMA (Robust Multichip Average) algorithm. Identification of differentially-expressed genes based on false discovery rates (FDR <0.05) indicated no statistical differences in gene expression among treatments (Criteria: at least ± 2-fold change and *P* <0.05). This is a common occurrence in complex biological experiments where there is high variation among treatments and low gene expression levels. Hence, we filtered differentially-expressed genes using only 2 criteria: i) an expression ratio of at least ± 2-fold change and ii) *P* < 0.05 in ANOVA tests comparing log_2_ (normalized hybridization intensity) of treatment to the mock control. We also validated microarray results using reverse transcription quantitative -PCR for genes of interest (described in detail below). The microarray experiment design details and raw microarray data is available at ArrayExpress under the accession number E-MEXP-3888 (http://www.ebi.ac.uk/arrayexpress/experiments/E-MEXP-3888/). Differentially-expressed genes were assigned functional annotations with Blast2GO software [45] and classified by GO-biological process and cellular component using default parameters and an *E*-value cut-off of 10^-6^.

### Principal components analysis of the microarray hybridization data

Principal components analysis was performed on fold-change expression for significantly-expressed genes to visualize general differences in global gene expression profiles among the TSWV and TSM treatments and to elucidate patterns of gene expression consistent with biologically informative processes involved in TSWV- and TSM-plant interactions. This pattern analysis approach is designed to reduce a large number of variables in a dataset, e.g., gene probes on a microarray, into a small number of components for the purpose of visualizing complex interactions among the original variables in a two or three-dimensional space [46]. Each principal component (PC) generated is a linear combination of all of the values in the dataset, and each PC successively explains the amount of variation in the dataset. Variables (gene probes and their corresponding expression values) that are correlated with one another and mostly independent of other variables are combined into each PC.

Global gene expression patterns for each of the three treatments relative to the mock control (i.e., +/- fold change of log_2_- transformed hybridization values) were compared separately. Probes of genes that were found to be differentially-expressed (*P* < 0.05) in at least one of the three treatments (TSWV, TSM, or TSWV + TSM) compared to the mock control were included in analysis. As such, 1,895 probes (i.e., loading variables) were included in the PCA. PCA was performed with JMP Genomics (SAS Institute, Cary, NC) using a covariance matrix to calculate PC scores for each treatment-biological replicate, and loadings (i.e., Pearson correlations (r) for pairwise comparisons between fold change of a particular probe and each PC score) were calculated in JMP Genomics. Loadings (r) greater than or equal to 0.75 (+/-) with a *P*-value < 0.1 were considered to contribute significantly to the variation among treatments. Analysis of variance was performed on the PC scores using the GLM protocol and Tukey’s pairwise comparison test in Minitab v14 to determine if there were general differences in expression profiles among treatments.

### Validation of microarray data using relative reverse transcription quantitative –PCR (RT-qPCR)

We targeted five genes associated with the SA, JA, and antiviral small-RNA-mediated gene silencing pathways: BGL2 and NIM1 (SA pathway), AOS and CI (JA pathway), and RNA-directed RNA polymerase 1 (RDR1). We chose elongation factor 1-alpha (EF1) as the internal reference gene for normalization because expression of this gene was found to be invariant to TSWV infection or TSM challenge in the microarray experiments and had been previously shown to be stably-expressed in Moneymaker tomato systemically-infected with *Tobacco rattle virus* [47]. Target and EF1 primer pair sequences, their corresponding melting temperatures, and real-time PCR efficiencies are indicated in Table 4. The normalized abundance of TSWV nucleocapsid (N) RNA compared to EF1 was also determined to estimate virus titer in leaf tissue using primers tested previously [31]. We selected one plant per treatment (mock included) per biological replicate (i.e., 24 RNA samples in total) of the greenhouse experiment that represented the average fecundity and/or TSWV symptom severity for a given treatment. Subsamples of total RNA isolated from leaflet tissue used in the microarray hybridization experiment were treated with DNase using the rigorous DNA removal procedure of the Turbo DNA-free kit (Applied Biosystems Inc, Carlsbad, USA) and cDNA was synthesized from 1 µg DNA-free RNA using the iSCRIPT cDNA synthesis kit (BioRad, Hercules, CA, USA). Real-time PCR master mixes were prepared using iQ sybr Green Mix (BioRad) according to manufacturer’s specifications and final reaction (20 µl) concentrations of 200 nM of each primer. Reactions were performed in duplicate using the iCycler iQ Thermal Cycler with a 96 x 0.2 ml reaction module and iCycler iQ software (Bio-Rad). The PCR cycling parameter included a 2-step amplification and melt protocol: 95°C for 3 mins, then 40 cycles of 95°C for 1 min and 55°C for 45s; then melt protocol of 55°C for 1 min then 80 cycles of 10 seconds each with 0.5°C increase in temperature at each cycle.

**Table 4:** Reverse transcription quantitative-PCR (RT-qPCR) primer pair sequences and corresponding PCR efficiencies.

**Primer name**	**Gene name/** **accession number**	**Primer sequence (5´–3´, forward/reverse**)	**PCR Efficiency**
AOS	Allene oxide synthase/AJ271093	ATCGTCTTATCGTGTTAGTATTC/ GATGATGATGGTGATTGTGAT	1.98
BGL2	Beta-1,3-glucanase/M80604	CTTGTTGGGCTTCTAATCC/ CTTGATCCGATGGTAAATTATTG	1.91
CI	Cathepsin D inhibitor protein/X73986	GCGTTAGGTGGTGATGTA/ GAATTGTAGGTCCATTAGTTGAT	1.97
EF-1	Elongation factor -1 alpha/X14449	GATTGGTGGTATTGGAACTGTC/ AGCTTCGTGGTGCATCTC	1.97
NIM1	Non-inducible immunity 1/ NM_001247629	GATAAGTCCTTGCCTCAT/	2.00
		AATGCTCTATGTATCCTCTT	
RDR1	RNA-directed RNA polymerase 1/ Y10403	GCGACCTTCACAAGAGAT/ TCATAATGCCACCACTAAGT	1.80
TSWV-N^a^	TSWV nucleocapsid gene/AF306490	GCTTCCCACCCTTTGATTC/ ATAGCCAAGACAACACTGATC	1.90

PCR efficiencies were calculated as 10^- 1 /slope^. ^a^ Primer sequences obtained from [31].

The relative abundance of target RNA was determined for each treatment compared to the mock control. The relative expression ratio (RER) equation [48] was calculated as follows: RER = E_target_^ΔCttarget[control-treatment^]/E_ref_^ΔCtref[control-treatment]^ where E refers to the PCR primer efficiency for target or internal reference (EF1) genes and ΔCt is the difference in Ct-values (i.e., threshold cycle values automatically calculated by the IQ software) between a treatment and mock control. The average Ct value (n = 3) obtained for the mock control for each target and reference gene was determined and used in the RER calculations. To estimate virus titer, the normalized abundance of TSWV N RNA (genomic and transcript RNA) was calculated using the Pfaffl inverse equation [48]: E_ref_^Ctref^ / E_N_^CtN^ as described previously [49].

### Phytohormone quantification

Plant hormones evaluated included salicylic acid (SA), jasmonic acid (JA), jasmonyl-isoleucine (JA-IL), and 12-oxo-phytodienoic acid (OPDA). Frozen tomato leaflet samples were sent to The Donald Danforth Proteomics & Mass Spectrometry Facility, St. Louis, MO for chemical extraction and liquid chromatography–electrospray tandem mass spectrometry using methods described by Pan and co-authors [50]. Analysis of variance was performed on log_10_-transformed phytohormone contents (ng analyte g fresh weight^-1^) in Minitab using a GLM that included Treatment and Biological Replicate as two fixed factors and their interaction term and greenhouse Block as the random factor. The analyses revealed no apparent main effect or treatment interaction for any of the phytohormones measured due to biological replicate; therefore, data obtained for the three biological replicates were pooled and one-way ANOVA was performed to determine the main effect of treatment on phytohormone contents. Pairwise treatment comparisons were performed using Tukey’s family error rate (*P* < 0.05).

### Total free amino acid quantification

Total free amino acid content in tomato leaf tissue samples was analyzed using a protocol described by Fahaam and co-authors [51] with a few modifications. Briefly, whole leaves were harvested from tomato plants and freeze-dried in an oven/ desiccator at 80°C for 2-3 days or until no change in weight was recorded. Approximately 0.1g of dry tissue was extracted with 10 ml of 70% hot ethanol and centrifuged at 2500*g* for 5 min. The dry residue was dissolved in 2.5 ml of 0.1 N HCl and kept at -20°C until assayed. Colorimetric procedures were adapted to Technicon Autoanalyzer II for simultaneous determination of total free amino acid content based on an internal standard (leucine) in plant tissue samples modified from [52]. Total free amino acid content data were analyzed using a statistical model similar to that described in the Phytohormone quantification section; however, biological replicate had a significant main or treatment interaction effect and therefore, data from biological replicates were interpreted separately.

## Supporting Information

Table S1 **Differentially-expressed genes in tomato plants systemically-infected with TSWV and/or infested with TSM.** Statistically significant (*P*< 0.05, fold change > ± 2).
(XLSX)

Table S2 **Differentially-expressed genes in tomato plants systemically-infected with TSWV.** Statistically significant (*P*< 0.05, fold change > ± 2).
(XLSX)

Table S3 **Differentially-expressed genes in tomato plants infested with TSM.** Statistically significant (*P*< 0.05, fold change > ± 2).
(XLSX)

Table S4 **Differentially-expressed genes in tomato plants systemically-infected with TSWV and infested with TSM.** Statistically significant (*P*< 0.05, fold change > ± 2).
(XLSX)
